# Forensic psychiatry, one subspecialty with two ethics? A systematic review

**DOI:** 10.1186/s12910-018-0266-5

**Published:** 2018-04-10

**Authors:** Gérard Niveau, Ida Welle

**Affiliations:** Geneva University Hospitals, Rue Gabrielle-Perret-Gentil 4, 1205 Geneva, Switzerland

**Keywords:** Forensic psychiatry, Ethics, Principlism, Consequentialism, Legal psychiatry, Correctional psychiatry, Autonomy, Beneficence, Non-maleficence, Justice

## Abstract

**Background:**

Forensic psychiatry is a particular subspecialty within psychiatry, dedicated in applying psychiatric knowledge and psychiatric training for particular legal purposes. Given that within the scope of forensic psychiatry, a third party usually intervenes in the patient-doctor relationship, an amendment of the traditional ethical principles seems justified.

**Results:**

Thus, 47 articles, two book chapters and the guidelines produced by the World Psychiatric Association, the American Association of Psychiatry and the Law, as well as by the Royal Australian and New Zealand College of psychiatrists, were analyzed. The review revealed that the ethics of correctional forensic psychiatry and those of legal forensic psychiatry do not markedly differ from each other, but they are incongruent in terms of implementation.

**Methods:**

In an effort to better understand which ethical principles apply to forensic psychiatry, a chronological review of the literature published from 1950 to 2015 was carried out.

**Conclusion:**

The ethics of correctional forensic psychiatry are primarily deontological. The principle of justice translates into the principle of health care equivalence, the principle of beneficence into providing the best possible care to patients, and the principle of respect of autonomy into ensuring confidentiality and informed consent. The ethics of legal forensic psychiatry are rather consequentialist. In this latter setting, the principle of justice is mainly characterized by professionalism, the principle of beneficence by objectivity and impartiality, and the principle of respect of autonomy by informed consent. However, these two distinct fields of forensic psychiatry share in common the principle of non maleficence, defined as the non collaboration of the psychiatrist in any activity leading to inhuman and degrading treatment or to the death penalty.

## Background

Like many other specialities in medicine, psychiatry is a broad discipline covering a various panel of subspecialties. Forensic psychiatry is a particular branch that emerged a few decades ago, and since then, its role has constantly risen in importance [1–5]. A definition of forensic psychiatry often cited is the one chosen by the American Academy of Psychiatry since its revision in 2005: « a subspecialty of psychiatry in which scientific and clinical expertise is applied in legal contexts involving civil, criminal correctional regulatory or legislative matters, and in specialized clinical consultations in areas such as risk assessment or employment» [6]. While having the advantage of being rather general, this definition lacks sufficient accuracy. The forensic section of the World Psychiatric Association lists the various functions of the forensic psychiatrist: assessment of mentally disordered offenders, expert witness in civil and criminal jurisdiction, advice to general psychiatrists and other professionals, as well as treatment of mentally disordered offenders [7]. Regardless of its specific field of application, forensic psychiatry is subject to ethical and moral conflicts, as it not only involves the traditional physician-patient relationship, but also includes the triangular relationship between the patient, the physician and the society, the latter represented by the judicial or penitentiary authority [6]. It is therefore essential that the forensic psychiatrist refers to solid ethical values and principles. Ethics is a discipline any physician should be capable to deal with according to his or her own conscience. Nonetheless the establishment of normative ethical principles is a precious guide to professionals confronted to moral dilemmas in their daily practice [8], and even more so in forensic psychiatry.

While considering moral judgments, and how moral beliefs are coordinated, Beauchamp and Childless concluded that four main principles may be used as guidelines in medical ethics [9]: the respect of autonomy, beneficence, non-maleficence and social justice. These authors insist on the fact that none of the four principles aforementioned should have priority over one another, and that their use in daily practice should depend on the particular case considered. The principist approach of Beauchamp and Childress, largely recognized and accepted worldwide, represents a platform for common reflection on clinical ethics [10]. However, the consequentialist approach is often mentioned as a means to solve ethical conflicting situations, and in which the principist approach is not satisfactory.

The aim of this paper is to identify, in an optic of normative ethical practice, which ethical priorities are the most relevant in the field of forensic psychiatry, through a bibliographical research.

## Methods

Bibliographical references were searched on Medline and Psycinfo database. The key word « ethics » was associated with the boolean operator « AND » to the following search terms: “forensic psychiatry”, “prison psychiatry”, “expert witness”, “legal psychiatry”. All texts between 1950 and 2015 were initially retrieved.

The texts that were excluded were non English language publications, texts evocating ethical questions as a secondary subject, and texts repeating previous authors’ conclusions. At the end 47 texts and two book chapters were retained for further analysis.

The search was completed by consulting the main professional associations on general psychiatry and forensic psychiatry. Ethical guidelines were retrieved on the World Psychiatric Association website, the American Association of Psychiatry and the Law, and the Royal Australian and New Zealand College of psychiatrists.

## Results

Forensic psychiatry is a subspecialty that emerged in the 1950s, evolving progressively and quite differently according to the countries concerned. Interestingly, the ethical questions pertaining to this subspecialty are relatively recent. As a matter of fact, until the 1960s, publications regarding the ethical questions in forensic psychiatry were rather scarce. It is only during the last 50 years that ethical guidelines became more structured. In order to take this evolution into consideration and to better understand the current opinions, results of the review of the literature are presented chronologically.

The first texts addressing the question of ethics in forensic psychiatry are those of Scott, Diamond and Bartholomew [11–14]. These authors mention specifically the question of confidentiality of the psychiatrist who intervenes as a therapist in a prison environment, and whose duty is to give impartial expert assessments to the court. Bartholomew recognizes the notion of « degrees » in applying professional secrecy, depending on the nature of this secrecy and the nature of medico legal situation being dealt with.

In 1969, a Committee on Ethics was created within the American Academy of Psychiatry and the Law (AAPL). The successive presidents of this committee reflected upon the creation of specific guidelines in forensic psychiatry [15]. A serious questioning on ethics in forensic psychiatry began only after Alan Stone’s contributions in 1980. Stone, who was at that time the president of the American Psychiatric Association, mentioned in his presidential speech « the parable of the Black Sergeant » [16]. By using this parable, he expressed his strong reluctance to include psychiatry in the judicial process, concerned that the psychiatrist would be involved in an inappropriate activity from an ethical standing point. Following the lecture given at the 1982 Annual Meeting of the AAPL, Stone’s critics nurtured a cascade of reactions, and generated a thorough reflection on ethics in forensic psychiatry. Stone stated that forensic psychiatry stands outside the ethical limits defined by the American Medical Association [17]. In Stone’s own words, psychiatry « prostitutes itself » because it may as well harm justice and profit to the patient, or on the opposite, deceive the patient and profit to justice, in the context of an adversarial system [17].

This statement generated the so-called « Stone-Appelbaum controversy », and led to a special issue in the Bulletin of American Academy of Psychiatry and the Law (BAAPL) published in 1984, dedicated to the question of ethics [17, 18]. In this bulletin, the text of Stone’s speech was published under the title « The ethical boundaries of forensic psychiatry: a view from the ivory tower » [17], and various authors replied to Stone. Appelbaum rejected the idea of an incompatibility between psychiatry and the judicial system, insisting that the quest of truth and even the objective truth should constitute the psychiatrist’s cornerstone of ethical principles at the court [18]. Weiner commented that Stone wished to apply the same ethical principles as those existing between the classical relationship patient physician, without considering the best interest of justice and society [19]. Halleck signalled the ethical problem of the psychiatrist playing the « double-agent role », assuming simultaneously the function of a therapist and that of an evaluator [20]. Ciccone and Clements considered that « the ethicists’ notion of rights and autonomy are not the relevant ethical issues » in the forensic domain, hence proposing the concept of « respect for individuals » [21]. They argued that the goals followed by psychiatrists working within the legal system should in no way differ from those applied by physicians practicing in the therapeutic field.

In the same year, Appelbaum stipulated that the question of confidentiality be treated the same way whether within the forensic field or in psychiatry in general, but with certain nuances to be added when other interests were at stake, such as for instance ensuring the protection of the public [22]. In his view, weighing the real interest in preserving secrecy of the person involved against the interest of society is crucial, and accordingly preserving the individual’s secrecy interests prevails above the interest of society.

An intense period of reflection took place in the United States in the middle of the 80s, on the positioning of forensic psychiatrists towards the death penalty. This reflection culminated with the case of John Hinckley Jr., who was declared not guilty by reason of insanity, after having shot and wounded President Ronald Reagan. This verdict had numerous implications for forensic psychiatry, and consequently the insanity defence was abolished in four US States. In 1986, the US Supreme Court declared that the Eighth amendment of the Constitution banned the execution of mentally ill [23]. Commenting on this decision, Appelbaum insisted on the importance of impartiality of the psychiatrist intervening as an expert in criminal cases {Appelbaum, 1987 #538]. In 1987, the AAPL published its first version of « Ethical Guidelines for the Practice of Forensic Psychiatry », insisting on applying the principles of (a) respect for confidentiality, (b) informed consent, (c) honesty and striving for objectivity, as well as (d) detaining the required qualifications. Radelet described the confrontation between these guidelines and the situation of mental patients sentenced to death as an “ethical chaos” [24]. However, the issue of treatment and the evaluation of death-sentenced criminals were held in abeyance.

One of the main works of Appelbaum in the field of ethics published in 1990 is a clear response to Stone’s statements cited 6 years earlier [25]. Appelbaum declares being favourable to a distinct ethical approach applied by the forensic psychiatrist who intervenes in court, as opposed to psychiatrists acting as therapists. He claims that the principles of beneficence and non-maleficence are not essential to the psychiatric expert, given that he is not involved in the therapeutic relationship with the patient. When defining the specific principles to be applied in forensic psychiatry, Appelbaum refers to the guidelines of the AAPL, however insisting on the importance of each physician to determine which principles are of most relevance, depending on the situation in question.

In 1992, Appelbaum published a paper on the ethical implication regarding the evaluation of dangerousness [26]. He reminds that the prediction of dangerousness cannot be objective, as it does not rely on a scientific basis, and describes this process as unethical. Appelbaum insists on the importance of integrating the context into the ethical dimension. Certain circumstances justify that the psychiatrist evaluates the dangerousness without hurting the ethical principles of non-maleficience.

In 1996, the World Psychiatric Association approved the Madrid Declaration on Ethical Standards for Psychiatric practice [27]. In the guidelines concerning specific situations, the declaration states that “psychiatrists shall not take part in any process of mental or physical torture”, “nor participate in assessments of competency to be executed”. Furthermore, the guidelines state that “it is the duty of a psychiatrist with dual obligations and responsibilities (…) to disclose to the person being assessed the nature of the triangular relationship and the absence of a therapeutic doctor-patient relationship” [27].

In 1997, Appelbaum published an essential paper on the ethics in Forensic Psychiatry [18]. Placing himself exclusively from the perspective of the psychiatric expert at court, he showed the significance of the principles of truth-telling and respect for persons. According to Appelbaum, the distinct ethical positioning of the forensic psychiatrist is the basis for distinguishing his role as a therapist or as an expert, and it is therefore essential to leave behind the theory of a « mixed model », which poses the significant « double agent problem » initially identified by Stone.

In 1998, Griffith made an original contribution by proposing to re examine the question of ethics in the forensic field, taking into account specific characteristics of social minority groups [28]. At the end, he ended up not proposing any specific guideline for this particular group.

In the third edition of Psychiatric Ethics published in 1999, Gutheil examined the situation of the psychiatrist intervening as an examinator and not as a therapist [29]. He emphasized on the particularities related to managing the confidentiality and of consent by the individual, as well as the core role of objectivity and honesty.

In Europe, Gunn rejected the idea that a different ethic could be considered depending on the role the physician plays [30]. He writes: « for my part, I find it very difficult to understand how a doctor can stop being a doctor ». Gunn dismissed the notion to « tell the truth » as a reference ethic for the forensic psychiatrist. On the same line, Nedopil considered that the forensic psychiatrist does not violate the ethical principles of medicine as long as he remains within the limits of his role and of his knowledge as a psychiatrist [31]. He considers that Stone’s critics should be reconsidered in the light of the present knowledge in the field of evaluation of dangerousness and the relationship between crime and mental health.

In 2003, the Royal Australian and New Zealand College of Psychiatrists adopted the ethical guidelines nr. 9 concerning « Ethical guidelines for independent medical examination and report preparation by psychiatrists». The overriding principles in the field of forensic psychiatry stated in these guidelines are the same as those described by the AAPL: honesty and strive for objectivity [32].

In the United States of America, the opinion that a specific set of ethics applied to experts in forensic psychiatry, as opposed to conventional forensic therapeutics, gradually consolidated itself. In 2004, Bailey et al. defined the following four ethical priorities in forensic psychiatry: « (a) respect for the individual’s right to privacy and the maintenance of confidentiality; (b) the need to obtain the informed consent from the individual before undergoing the forensic evaluation; (c) the adherence to the principles of honesty and striving for objectivity; d) have a sufficient experience and qualifications (…) » [33].

According to Adshead and Sarkar, the two principal ethical principles aimed at guiding the forensic psychiatrist should remain « beneficence » and « respect of justice » [34]. There is indisputably a tension between these two principles in the field of forensic practice. Depending on the orientation taken, whether towards prison psychiatry or detaining the role of expert for the court, one of the principles clearly dominates over the other.

In 2005, the American Academy of Psychiatry and the Law (AAPL) adopted a new version of the Ethics guidelines for the practice of forensic psychiatry. These guidelines deal with the implementation of the general principles of medicine in the context of forensic psychiatry, in its therapeutical and evaluation aspects [6]. The ethical guidelines stated by the AAPL are, once again, respect of confidentiality, informed consent, honesty and striving for objectivity, and qualifications.

The major development of forensic psychiatry by the end of the twentieth century brought the World Psychiatric Association to devote an entire issue of its journal to this subject in 2006. Arbolada-Florez sees in the dual specificity of forensic psychiatry, medicolegal and psychiatric, the foundation of its ethical priorities [35]. According to him, it is highly relevant that the forensic psychiatrist clarifies his role to the individual being treated or evaluated. As an evaluator, he is required to act neutrally and can therefore not guarantee that all his actions be to the favour of the individual [14]. On the other side, as mentioned in the book by Candilis et al., the role of the evaluator implies that the psychiatrist has an ethical obligation towards the individual under evaluation in explaining how his conclusions are motivated and its consequences [36]. In any case the forensic psychiatrist must never participate in any acts of torture or similar handlings that are contrary to human rights. This view is supported by different authors who treated the ethical aspects in the special issue of World Psychiatry [5, 37–39].

Also in 2006, Jager justifies that ethical principles in forensic psychiatry have the peculiarity of asking the physicians in this specialty to take a dual responsibility that is, towards the individuals but also towards society in general [5]. That same year, Taborda et Arbolada-Florez published a lengthy paper covering the differences in ethical priorities in the case of the expert psychiatrist and that of the prison psychiatrist [2, 35, 40]. These authors insist on the positioning of the evaluating psychiatrist towards the expertised individual, and the obligation of only intervening in the area of one’s own competencies. They underscore that the expertised must be clearly informed on the expert’s role and give his informed consent to participate in the evaluation. The most important ethical aspect is still the expert’s impartiality. For this reason, there is a « profound ethical incompatibility between medical treatment of a patient and giving an expert opinion regarding that same individual ». On the opposite, the psychiatrist who intervenes in the treatment of individuals in a correctional setting is committed to the aforementioned ethical principles of confidentiality and respect of autonomy. However, these principles need to be adapted to the context of prison environment, particularly because of the question of “dual mandate” regarding the practice of psychiatry in a correctional setting.

The idea expressed by Birmingham, Wilson and Adshead regarding prison psychiatry is quite similar, but they insist on the ethical necessity that patients in prison be provided with the same quality of care compared to the general population [41]. This point of view discards the concept that the prison psychiatrist detains a specific role that amends the implementation of fundamental ethical principles.

According to Sen, Gordon, Adshead et al., the difficulty in implementing the « four principles plus scope » in a forensic context, results from the obligation to consider a third party, represented by the society in general [10]. In the rise of ethical conflicts in forensic psychiatry, it is the principle of justice that prevails, contrary to the principle of autonomy that is less relevant in this particular field of psychiatry.

In a consensus paper published in 2007, Konrad et al. places the principle of equivalence of care at the centre of ethical questions in prison psychiatry [42]. During the same year, Gordon et Lindqvist evaluated the progress made in forensic psychiatry in Europe [43]. They considered that the psychiatric expert at court should be impartial, while still remaining « concerned for the welfare of the offender ». They stipulated that the Human rights guaranteed by the European Court of Human Rights, should provide a benchmark regarding ethics of care in prison psychiatry.

The year 2008 was marked by the publication of a special issue in the Journal of AAPL dedicated to a retrospective on the evolution of ethical concepts in forensic psychiatry since the speech given by Stone at the Annual Meeting of the American Academy of psychiatry and the Law de 1982. Three dimensions were profiled: Appelbaum confirmed his vision on an ethic centred on truth-telling and respect of persons [44], and Griffith his view on the relevance that forensic psychiatry considers the sociocultural position of the individual being evaluated [45]. Morse endorsed the ethical positions set by Appelbaum while insisting on the limits of the role taken by the psychiatric expert or the psychologist in the legal process [46]. Candilis’s approach was not that different from that of Appelbaum, but he stressed the need that the forensic psychiatrist recognizes the vulnerability of the subjects being evaluated, is fully conscious of his role and his position, and ensures he is honest and professional in his expert work at court [47].

In 2010, the World Psychiatric Association’s section of forensic psychiatry adopted a consensus paper on guidelines for independent medical examination [48]. The ethical principles listed in the position paper are acquiring an informed consent by the person to be evaluated, the absence of bias in the written report, the honesty and impartiality of the work, the expert’s diligence, and the respect for confidentiality in the legal framework.

That same year, Tataru et al. describe forensic psychiatry in Western Balkan countries as a young specialty, recognized since 2005 or 2007, relating to the court-ordered assessment and treatment « of people with mental disorder who show antisocial or violent behaviour» [49]. They maintain that the ethical questions remained in dispute, and they relate in their practice to the principles of Human Rights. Konrad, on the contrary, insists on the necessity to aim towards beneficence for the patient and the respect of equivalence of care in prison, including the consent in treatment and confidentiality [48]. Concerning the last-mentioned matter, Pinta discusses the limits of the principle of confidentiality in prison, in cases similar to Tarasoff duties, where the potential victim is imprisoned or in freedom [50]. Calcedo-Barba incorporates the concept of objectivity as proposed by Appelbaum, the ethical basis for forensic psychiatry [38]. He confronts it to the questions raised by the theoretical orientations of the DSM-5. He thereby concludes that the standard ethical position of objectivity is probably too optimistic, and to him seems rather illusory.

In 2013, Cervantes and Hanson assessed that it is not uncommon that psychiatrists intervene both as a psychotherapist and as an evaluator, giving rise to dual agency conflicts [51]. They underscore the ethical principles that should guide psychiatrists’ acts in the prison setting, namely beneficence, non-maleficence, neutrality, objectivity and justice. Similarly, Sakelliadis et al. and Trestman base themselves on the values of protection of human rights and identify equivalence of care, respect of confidentiality, informed consent and ability to refuse treatment, as the core ethical principles of practicing forensic psychiatry [52–54].

In 2014, the AAPL revised the Guidelines on Forensic Psychiatric Evaluation of Defendants raising the insanity defence [55]. In the new version of the Guidelines the specific ethical principles to the practice of forensic psychiatry were recalled. It included specific competence in the area of forensics, honesty and objectivity, respect and confidentiality, information and informed consent of the individual under evaluation. It also states that forensic psychiatrists need to be free of conflict of interests, and should not carry the double role towards the evaluated individual, in particular not be a therapist.

Concomitantly, Combalbert et al. underline the lack of clear directives in France for forensic mental assessments ordered by the court [56]. These authors emphasize on the necessity to improve the objectivity and the impartiality of forensic psychiatrists, when they intervene as experts in the court.

Forensic psychiatrists dealing with children and adolescents encounter an even greater difficulty, according to a statement by Kaltiala-Heino and Eronen in 2015 [57]. These authors note that the dual role of the forensic psychiatrist is aggravated by the immaturity and the position of dependence of the minor. Consequently, the ethical principles of beneficence, non-maleficence, respect of autonomy and justice are considerably put to challenge, in the best interests of the individual and society.

Recently, Buchanan reworked on the question of respect for the individual in forensic psychiatry, and proposed to enlarge it to the principle of respect for dignity, in its dimension of protection of the vulnerable individuals [58].

## Discussion

### What is forensic psychiatry?

According to Gutheil, the word « forensic » stems from the latin « forum » and makes reference to the court [29]. Forensic psychiatry would have its origins among psychiatrists intervening at the court. However for many years, prison psychiatry was perceived as a form of forensic psychiatry. Several authors [30, 35] have pinpointed the fundamental differences underlying these two fields: on one side forensic psychiatry in the correctional setting, often named correctional psychiatry or prison psychiatry, is defined as a therapeutic field aimed at giving treatment to offenders accused or condemned of a criminal act, imprisoned or maintained in freedom. On the other side, forensic psychiatry for the court, or legal psychiatry, is a field of evaluation consisting in producing a report for the penal or civic authorities, and includes the role of expert witness. This distinction between the roles of expert at court and that of therapist for the accused and the condemned only took place between the middle and the end of the twentieth century. The history of forensic psychiatry is, according to Engstrom, “like the contemporary specialty itself, truly a mixtum composition” [3]. Forensic psychiatry in the United Kingdom is essentially understood as prison psychiatry, while in the United States it is first of all defined as psychiatry for the court [2, 8, 40]. In continental Europe and in Latin countries, the term forensic psychiatry frequently covers both duties, that of a therapist and of a forensic expert. Furthermore, the World Psychiatric Association includes the therapeutic functions and the expert functions in its definition of forensic psychiatry [59]. There is a constant ongoing debate focusing on whether forensic psychiatry should remain a medical profession or whether it should be limited to collaborating as an expert witness in a trial, as an independent service provider for the justice system. Let us not forget that medicine provides us with a body of knowledge and with a methodology that helps maintain our medical ethics, regardless of our specialty area [60]. We have reached a time period in history where the scientific “know-how” culture is considerably brought forward. In this perspective, the principles of justice, social interests, non-maleficence are value systems that tend to overlook autonomy and beneficence for the individual [61]. Various medical professionals assessing individuals in compensation cases, working for insurances or public services or for the prevention on infectious diseases are confronted with this dilemma. Decisions are mainly based on valuing the individual based on his general function, how he can contribute to the society in general, rather than the individual’s condition and autonomy. This gives rise to considerable debate on how to make good-quality ethical decisions in cases where people cannot express their own views.

Clearly, the role of a therapist and that of an evaluator for the judicial authority are incompatible. In 2011, Christopher et al. showed in a comparative study the importance of not fulfilling both duties towards the same individual, even in the civil domain, in order not to be drawn into a dual-agency conflict [62]. Depending on the type of function he fills, the position of the forensic psychiatrist towards the patient and towards society is not similar [18, 63]. As a consequence, his ethical priorities are not identical [34].

Psychiatry towards offenders with or without mental disorders can be carried out in different situations: ambulatory treatment within prison, special wing within the prison, specialized security hospitals, contractual arrangements with outside psychiatric facilities and forensic community corrections [35]. The specificity of these means of practice in psychiatry is characterized by the particular setting in which they take place. Therefore, the ethical principles of medicine and psychiatry should be adapted to this particular setting. The correctional forensic psychiatrist should be able to refer himself constantly with respect to the patient and to society.

Legal psychiatry addresses all situations in which a psychiatrist gives an opinion to a third authority and does not intervene at the request of the patient himself. The most spectacular role of the legal psychiatrist is that of his intervention at the criminal court regarding responsibility, dangerousness and measures for preventing the risk of recidivism. However, the role of the psychiatrist is also important in the civil area, concerning decisions relating to child custody, civil commitment, guardianship or competence at the time of writing a testimony [55].

The implementation of the four ethical principles considered as fundamental in the domain of forensic psychiatry are summarized in Table 1 and discussed below.

**Table 1:** Implementation in correctional psychiatry and in legal psychiatry of the four basic ethical principles of Beauchamp and Childress

	Correctional psychiatry	Legal psychiatry
Beneficence	Care in the best patient’s interest	Impartiality
Respect for autonomy	Informed consent and respect of confidentiality	Informed consent
Justice	Equivalence of care	Qualifications
Non-maleficence	No participation to inhuman or degrading treatment or to death penalty	

#### Justice

The principle of justice stipulates that patients in similar conditions should have access to quality health care on an equal basis. Individuals subjected to forensic psychiatric intervention represent a particular vulnerable subgroup, compared with the general population. Thus, the main ethical question to be addressed first and foremost in this context is that of justice, with respect to forensic psychiatric intervention.

Legal sanctions can deprive individuals of their rights to come and go at will, but they do not reduce the right to access to health, at least in democracies. Health services developed in a correctional setting cannot accept to be used as a means of repression. For this reason, the majority of countries worldwide conforming to detention conditions in compliance with international human rights law adhere to the principle of equivalence in prison health care [37, 42, 48, 64, 65]. Various international conventions remind us of this principle of equivalence of health care given to detainees, in comparison to the community in general: European Convention on Human Rights, United Nations International Resolutions concerning Standard Minimum rules for treatment of prisoners, Recommendation N°R (98) 7 of the Council of Europe on the Ethical and Organizational aspects of health care in prisoner, Declaration of Tokyo of the World Medical Association, Declaration of Hawaii of the World Psychiatric Association.

However, the quality of care offered to detainees is constantly being challenged by a coercive setting and their vulnerable condition [66]. It is therefore crucial that forensic psychiatrists refer, in their daily practice, to the principle of equivalence of health care, stating that all individuals of a given community be provided with the same basic healthcare, independent of their social status.

On the opposite, in legal forensic psychiatry, the question of equivalence in healthcare is irrelevant. The issue here is not how to best serve the individual who is being evaluated, but how to use the psychiatrist’s skills most competently to answer the questions raised in the legal setting [19]. Viewed in a consequentialist perspective, the principle of justice translates into implementing equal means to evaluate individuals subjected to law. This equality can only be achieved if the expert has the required competencies to carry out his mission [67, 68]. The ethical principle of justice, applied in the field of forensic legal psychiatry, claims that the forensic psychiatrist fulfils his mission in a thorough and competent manner.

#### Beneficence

Beneficence is often cited as the most important ethical principle in medicine [69]. In general psychiatry, the principle of beneficence can easily be in tension with the principle of non-maleficence in situations such as non voluntary hospitalisations or treatments given under constraint. In forensic psychiatry, the same ethical conflicts may occur but increase in complexity due to the physical constraints present in the correctional environment.

For security purposes, the judicial or the correctional authority is often called upon to impose a treatment to a patient. However, the principle of beneficence holds that it is the forensic psychiatrist who recommends the best treatment for the health of the patient. It is not his duty, in principal, to impose a treatment that has been mandated by the authorities. This makes the practice of psychiatry all the more difficult in a correctional environment. As such, the principle of beneficence is present in the same legal way as it is in general psychiatry, but in a more constraining setting and can be viewed as potentially conflicting.

When intervening at court, the role of the forensic psychiatrist differs substantially because he does not act as a therapist [70]. This depiction of legal forensic psychiatry is narrow, as it does not relate to reality in practice. For instance, the legal psychiatrist enables mentally ill individuals not to be condemned as delinquents, dangerous detainees not to be set free, and those incapable of working to benefit from a disability pension [19]. Indeed, it cannot be refuted that the action of the legal psychiatrist may consequently result in a number of detainees being maintained in security facilities. If the legal psychiatrist remains in logic of deontological ethics, he can perceive his action as unethical. Whereas by accepting a utilitarian logic, he realizes that his action is clearly in agreement with the principle of « the greatest good for the greatest number ». The ethics of beneficence consist in adopting an impartial attitude, allowing valuing equally the interests of the individual and the interests of society. Furthermore, giving more prominence to public protection may be viewed as non ethical because of the negative consequences to the person under trial. On the opposite, assessing a favourable statement would equally be seen as non ethical due to possible harmful consequences for the public security as well as for the convicted individual. Gordon and Lindqvist consider that the question of impartiality has been recognized for more than 50 years in Great Britain as being a core component in legal psychiatry, following the paper by Scott in 1953 [43]. Nevertheless, certain judicial systems may place the psychiatric expert in substantially different Medico-Legal positions. In an adversarial system, the expert is engaged by one of the parties, while in the inquisitorial system the expert is engaged by the State. Weiner and Stone mean that the fact that some experts are named as “defence psychiatrists” while others as “prosecution psychiatrists” can be considered as “to prostitute the profession” [17, 19]. In fact, numerous intermediate systems of justice do exist, and the psychiatrist’s position may vary accordingly. This entails the need for the expert to refer to the ethical position of his profession, and not to the legal system. The expert’s ethical priorities need to be defined independently of the type of judicial system in which he intervenes. In this respect, the expert’s impartiality is a universal principle [61]. Consequently, the conclusions of the expert must not depend on who paid the fees of the psychiatrist, nor should they be affected by the type of acts committed, the personal history of the psychiatrist, or the risk of criminal conviction. Weiner points out that it is not the psychiatrist’s responsibility to “make the case”. He or she is in court to present his or her findings, « no more, no less ». Thus, in the light of a democratic and fair judicial system, the principle of beneficence translates into a rigorous practice of impartiality in the field of forensic psychiatry.

### Respect for autonomy

Autonomy is a highly valued deontological principle in our western societies. Respecting the autonomy of the patient has become a major ethical principle in numerous medical disciplines, following the rejection of medical paternalism. It should however be pinpointed that the question of autonomy and that of free consent differ considerably, whether in correctional forensic psychiatry or in legal forensic psychiatry.

In a correctional setting, the question of respect for autonomy is particularly sensitive. For instance, incarcerated individuals, or those detained involuntarily for treatment of a mental disorder may find themselves in a vulnerable position, following the restriction by the justice of their autonomy of decision [48]. Nonetheless, the domain of strictly personal rights, more specifically, the rights related to body integrity and to health, are not concerned by this reduction of decisional autonomy. This implies the condition that the forensic psychiatrist does not exploit the vulnerability of the patient under captivity, by imposing medical decisions [71]. The risk of deviation from the role of physician is high if the willingness of justice or the best interest of society is privileged. Under no circumstances should the psychiatrist engage as an agent of social control. Independent of the degree of restriction or reduction of freedom, the autonomy of the patients in the domain of health should be preserved. Respect for autonomy is concretely expressed within two domains often questioned namely the right to free and informed consent to treatment, and the respect of confidentiality. In a correctional setting, the principle of respect for autonomy is in tension with the principle of beneficence. As an example, the free choice of treatments and performed tests is relatively often thwarted by the obligation of treatment issued by the justice or by the necessities of community life in prison. Seen in this perspective, autonomy is constantly under challenge due to the need to take into account considerations of social justice. Similarly, respect of confidentiality, which is required to ensure a trusting relationship between the detainee and the health professional in order to maintain, may find its limits when safety and security is in danger [66, 72].

In forensic legal psychiatry, the forensic psychiatrist is not a care giver and the principle of respect of autonomy does not concern the consent to treatment or the respect of confidentiality. Undoubtedly, all persons subjected to an evaluation requested by the authorities should be provided with the freedom of choice to participate or not. Furthermore, it is the duty of the forensic psychiatrist to inform the person being evaluated of the specificities of his role, such as the absence of therapeutic function, and that the obligation of professional confidentiality is not applied in relation to the judicial authority [73, 74]. It is crucial that the individual under evaluation understands that this process may have negative consequences on him/her. A person who has not been given sufficient information regarding his rights will lose confidence in medicine and in justice in general. The application of the principle of autonomy in legal forensic psychiatry is an extension of the informed consent doctrine [61]. This principle, regarded as far more deontological than utilitarian, takes a consequentialist dimension, by enabling the subject to consent or to refuse, in the full knowledge of the expertise [18, 75].

#### Non-maleficence

The principle of non-maleficence is known as one of the most ancient ethical principles in medicine and is deeply rooted in medical practice [69]. In the correctional setting this principle is challenged due to the vulnerability of the relevant population. On many occasions, the psychiatrist must weigh arguments in favor of beneficence against those that may be considered maleficent, in the sense that it benefits the interest of others rather than the patient himself. This concerns particularly situations such as hunger strikes or activities for the purpose of research [10, 72]. In some countries, the principle of non-maleficence should protect the forensic psychiatrist against engaging in acts which are morally illicit and dictated by government representatives [76]. Numerous authors insist on the fact that the forensic psychiatrist must not take part in inhuman or degrading procedures, and in particular acts amounting to the death penalty [77].

With regards to psychiatry for the Court, Stone introduced as early as 1980, during his American Psychiatric Association Presidential Address, the ethical question of the engagement of the forensic psychiatrist in the activities of the Court. He formulated it as « The Parable of the Black Sergeant ». Stone questioned whether a physician may accept to participate in a judicial process where the final decision appears inequitable and immoral. This debate is recurrent in a number of American publications [78], emphasizing on psychiatrists taking part in a judicial system allowing the death penalty. More generally, this concept questions the appropriateness of psychiatrists in participating in legal systems that recognize inhuman and degrading treatments on persons. Clearly, the implication of a physician to such treatments, regardless of the type of relation he keeps with the concerned person, is contrary to the core ethical principle of the respect for human dignity, integrity and life [69]. It is the responsibility of each physician to reflect on the limit beyond which a treatment imposed by a government is inhuman and degrading, considering torture and death as unbreakable end-points. The ethical stance of the psychiatrist needs to be clearly defined. By renouncing in any involvement with the judicial system, based on the existence of inhuman and degrading sanctions, the mentally ill are left with the risk of being no longer protected against such sanctions. On the other hand, engaging in such procedures constitute a grave professional misconduct for a physician [58].

Legal psychiatry and correctional psychiatry both share the ethical perspective of non-participation in acts involving inhuman and degrading treatment, including that of death penalty. This ethical standpoint probably stems from the principle of conscientious objection.

## Conclusions

We have come to understand that the fundamental ethical values of medicine can and must be applied in forensic psychiatry, but their application in prison psychiatry and in legal psychiatry differ from each other in terms of significance and aim. Beauchamp and Childress insist on the fact that there is no order of priority for implementing the principles, as their relative importance depends on the specific situation. In forensic psychiatry, the principles proposed by Beauchamp & Childress retain their full strength, but the meaning and application of the ethical principles differ considerably, depending on whether they are applied in the field of prison psychiatry or legal psychiatry. In prison psychiatry, the physician is challenged by the institutional environment, and must therefore refer to a strong and legally enforceable code of conduct based on the principle of justice, and ensure that the principle of equivalence of care is respected. In legal psychiatry, the principle of beneficence is of outmost importance, in the form of impartiality by the expert’s actions in the court.

From an ethical perspective, a psychiatrist may satisfy both functions of legal forensic and correctional forensic psychiatrist, but cannot fulfill simultaneously the two duties on the same individual [18, 63]. Legal Psychiatry and correctional psychiatry are distanced from one another by ethical principles. And yet, legal psychiatry and correctional psychiatry join each other under one common ethical aspect: the principle of non-participation in inhuman, degrading actions, and the death penalty.

## References

[1] Abdalla-Filho E, Taborda JG (2006). The rebirth of forensic psychiatry. Rev Bras Psiquiatr.

[2] Arboleda-Florez JE (2006). The ethics of forensic psychiatry. Curr Opin Psychiatry.

[3] Engstrom EJ (2009). History of forensic psychiatry. Curr Opin Psychiatry.

[4] Gold HL (2005). A subspeciality of growing importance. Psychiatr Times.

[5] Jager AD (2006). Forensic psychiatry: a developing subspecialty. World Psychiatry.

[6] American Academy of Psychiatry and the Law. Ethics guidelines for the practice of forensic psychiatry adopted May 2005.Bloomfield, CT.:[http://www.aapl.org/docs/pdf/ETHICSGDLNS.pdf]; Accessed 12 Dec 2017.

[7] Association WP (2015). Introduction to forensic psychiatry for medical students.

[8] Gutheil TG, Bloch S, Green SA (2009). Ethics and forensic psychiatry. In: psychiatric ethics.

[9] Beauchamp TL, Childress JF (2009). Principles of biomedical ethics.

[10] Sen P, Gordon H, Adshead G, Irons A (2007). Ethical dilemmas in forensic psychiatry: two illustrative cases. J Med Ethics.

[11] Scott PD (1953). Psychiatric reports for magistrates courts. British J Delinquency.

[12] Bartholomew AA (1970). Confidentiality and the forensic psychiatrist in the correctional setting. Aust N Z J Psychiatry.

[13] Bartholomew AA (1966). Some problems of post-conviction--pre-sentence reports with particular reference to subsequent psychiatric treatment and rehabilitation. Med J Aust.

[14] Diamond BL, Weinhofen H (1953). Privileged communication and the clinical psychologist. J Clin Psychol.

[15] Weinstein HC (1984). How should forensic psychiatry police itself? Guidelines and grievances: the AAPL Committee on ethics. Bull Am Acad Psychiatry Law.

[16] Stone AA (1980). Presidential address: conceptual ambiguity and morality in modern psychiatry. Am J Psychiatry.

[17] Stone AA (1984). The ethical boundaries of forensic psychiatry: a view from the ivory tower. Bull Am Acad Psychiatry Law.

[18] Appelbaum PS (1997). Ethics in evolution: the incompatibility of clinical and forensic functions. Am J Psychiatry.

[19] Weiner BA (1984). Ethical issues in forensic psychiatry: from an attorney's perspective. Bull Am Acad Psychiatry Law.

[20] Halleck SL (1984). The ethical dilemmas of forensic psychiatry: a utilitarian approach. Bull Am Acad Psychiatry Law.

[21] Clements CD, Ciccone JR (1984). Ethics and expert witnesses: the troubled role of psychiatrists in court. Bull Am Acad Psychiatry Law.

[22] Appelbaum PS (1984). Confidentiality in the forensic evaluation. Int J Law Psychiatry.

[23] Sullivan S. Ford v. Wainwright: states cannot execute insane - but how is insanity determined. John Marshall Law Rev. 1987;20

[24] Radelet ML, Barnard GW (1988). Treating those found incompetent for execution: ethical chaos with only one solution. Bull Am Acad Psychiatry Law.

[25] Appelbaum PS (1990). The parable of the forensic psychiatrist: ethics and the problem of doing harm. Int J Law Psychiatry.

[26] Grisso T, Appelbaum PS (1992). Is it unethical to offer predictions of future violence?. Law Hum Behav.

[27] World Psychiatric Association (1996). WPA Madrid declaration on ethical standards for psychiatric practice.

[28] Griffith EE (1998). Ethics in forensic psychiatry: a cultural response to stone and Appelbaum. J Am Acad Psychiatry Law.

[29] Gutheil TG (1999). The forensic psychiatrist as expert witness in malpractice cases. J Am Acad Psychiatry Law.

[30] Gunn J (2000). Future directions for treatment in forensic psychiatry. Br J Psychiatry.

[31] Nedopil N (2009). The role of forensic psychiatry in mental health systems in Europe. Crim Behav Ment Health.

[32] Gaughwin PC (2004). A consideration of the relationship between the rules of court and the code of ethics in forensic psychiatry. Aust N Z J Psychiatry.

[33] Bailey RK, Scarano VR, Varma S (2004). The practice of forensic psychiatry: is it the practice of medicine?. Am J Forensic Psychiatry.

[34] Adshead G, Sarkar SP (2005). Justice and welfare: two ethical paradigms in forensic psychiatry. Aust N Z J Psychiatry.

[35] Arboleda-Florez J (2006). Forensic psychiatry: contemporary scope, challenges and controversies. World Psychiatry.

[36] Candilis PJ, Layde JB (2007). Professional development in forensic psychiatry: the role of the American Academy of psychiatry and the law. Acad Psychiatry.

[37] Konrad N (2006). Forensic psychiatry in dubious ascent. World Psychiatry..

[38] Calcedo-Barba A (2010). Objectivity and ethics in forensic psychiatry. Curr Opin Psychiatry..

[39] Sharma BR (2006). Forensic considerations of surrogacy -- an overview. J Clin Forensic Med.

[40] Arboleda-Florez J, Weisstub DN (2006). The scope of forensic psychiatry: ethical responsibilities and conflicts of values. Sante Ment Que.

[41] Birmingham L, Wilson S, Adshead G (2006). Prison medicine: ethics and equivalence. Br J Psychiatry.

[42] Konrad N, Daigle MS, Daniel AE, Dear GE, Frottier P, Hayes LM, Kerkhof A, Liebling A, Sarchiapone M (2007). International Association for Suicide Prevention Task Force on suicide in P. Preventing suicide in prisons, part I. Recomm Int Assoc Suicide Prev Task Force Suicide Prisons Crisis.

[43] Gordon H, Lindqvist P (2007). Forensic psychiatry in Europe. Psychiatr Bull.

[44] Appelbaum PS (2008). Ethics and forensic psychiatry: translating principles into practice. J Am Acad Psychiatry Law Online.

[45] Griffith EE (2008). Stone's views of 25 years ago have now shifted incrementally. J Am Acad Psychitry Law.

[46] Morse SJ (2008). The ethics of forensic practice: reclaiming the wasteland. J Am Acad Psychiatry Law.

[47] Candilis PJ (2009). The revolution in forensic ethics: narrative, compassion, and a robust professionalism. Psychiatr Clin North Am.

[48] Konrad N (2010). Ethical issues in forensic psychiatry in penal and other correctional facilities. Curr Opin Psychiatry.

[49] Tătaru N, Marinov P, Douzenis A, Novotni A, Kecman B (2010). Forensic psychiatry in some Balkan countries. Curr Opin Psychiatry.

[50] Pinta ER (2009). Decisions to breach confidentiality when prisoners report violations of institutional rules. J Am Acad Psychiatry Law.

[51] Cervantes AN, Hanson A (2013). Dual agency and ethics conflicts in correctional practice: sources and solutions. J Am Acad Psychiatry Law.

[52] Trestman RL (2014). DSM-5 and personality disorders: where did axis II go?. J Am Acad Psychiatry Law.

[53] Sakelliadis EI, Vlachodimitropoulos DG, Goutas ND, Panousi PI, Logiopoulou AP, Delicha EM, Spiliopoulou CA (2013). Forensic investigation of suicide cases in major Greek correctional facilities. J Forensic Legal Med.

[54] Sakelliadis EI, Goutas ND, Vlachodimitropoulos DG, Logiopoulou AP, Panousi PI, Delicha EM, Spiliopoulou CA (2013). The social profile of victims of suicide in major Greek correctional facilities. J Forensic Legal Med.

[55] American Academy of Psychiatry and the Law (2014). AAPL practice guideline for forensic psychiatric evaluation of defendants raising the insanity defense. J Am Acad Psychiatry Law.

[56] Combalbert N, Andronikof A, Armand M, Robin C, Bazex H (2014). Forensic mental health assessment in France: recommendations for quality improvement. Int J Law Psychiatry.

[57] Kaltiala-Heino R, Eronen M (2015). Ethical issues in child and adolescent forensic psychiatry: a review. J Forensic Psychiatry Psychol.

[58] Buchanan A (2015). Respect for dignity and forensic psychiatry. Int J Law Psychiatry.

[59] World Psychiatric Association (2015). Forensic psychiatry, consensus guidelines for independent medical examinations.

[60] Candilis PJ, Martinez R, Dording C (2001). Principles and narrative in forensic psychiatry: toward a robust view of professional role. J Am Acad Psychiatry Law.

[61] Calcedo-Barba A (2006). The ethical implications of forensic psychiatry practice. World Psychiatry..

[62] Christopher PP, Arikan R, Pinals DA, Fisher WH, Appelbaum PS (2011). Evaluating psychiatric disability: differences by forensic expertise. J Am Acad Psychiatry Law.

[63] Strasburger LH, Gutheil TG, Brodsky A (1997). On wearing two hats: role conflict in serving as both psychotherapist and expert witness. Am J Psychiatry.

[64] Konrad N, Welke J, Opitz-Welke A (2012). Prison psychiatry. Curr Opin Psychiatry.

[65] Daigle MS, Daniel AE, Dear GE, Frottier P, Hayes LM, Kerkhof A, Konrad N, Liebling A, Sarchiapone M (2007). International Association for Suicide Prevention Task Force on suicide in P. Preventing suicide in prisons, part II. Int Comparisons Suicide Prev Services Correctional Facilities Crisis.

[66] Niveau G (2007). Relevance and limits of the principle of “equivalence of care” in prison medicine. J Med Ethics.

[67] Nedopil N, Gunn J, Thomson L (2012). Teaching forensic psychiatry in Europe. Crim Behav Ment Health.

[68] Nedopil N, Taylor P, Gunn J (2015). Forensic psychiatry in Europe: the perspective of the Ghent group. Int J Psychiatry Clin Pract.

[69] Beauchamp TL, Childress JF (2013). Principles of biomedical ethics.

[70] Sakelliadis EI, Spiliopoulou CA, Papadodima SA (2009). Health care provision in prisons: a review on European and international guidelines. Acta Clin Belg.

[71] Austin W, Goble E, Kelecevic J (2009). The ethics of forensic psychiatry: moving beyond principles to a relational ethics approach. J Forensic Psychiatry Psychol.

[72] Elger BS, Handtke V, Wangmo T (2015). Informing patients about limits to confidentiality: a qualitative study in prisons. Int J Law Psychiatry.

[73] Rappeport JR (1982). Differences between forensic and general psychiatry. Am J Psychiatry.

[74] Waithe ME, Rappeport JR, Weinstein HC, Baumrin BH (1982). Ethical issues in the practice of forensic psychiatry. J Psychiatry Law.

[75] Taborda JG, Arboleda-Florez J (2006). Forensic psychiatry ethics: expert and clinical practices and research on prisoners. Rev Bras Psiquiatr.

[76] Abramowitz MZ (2005). Prisons and the human rights of persons with mental disorders. Curr Opin Psychiatry.

[77] Trestman RL (2014). Ethics, the law, and prisoners: protecting society, changing human behavior, and protecting human rights. J Bioeth Inq.

[78] Weinstock R (2013). Commentary: the forensic report--an inevitable nexus for resolving ethics dilemmas. J Am Acad Psychiatry Law.

